# Numerical study and optimization of thermal environment regulation in poultry house ventilation systems

**DOI:** 10.1016/j.psj.2025.105786

**Published:** 2025-09-03

**Authors:** Changzeng Hu, Lihua Li, Yuchen Jia, Zongkui Xie, Yao Yu, Limin Huo

**Affiliations:** a College of Mechanical and Electrical Engineering, Hebei Agricultural University, Baoding 071000, China; b Key Laboratory of Broiler/Layer Breeding Facilities Engineering, Ministry of Agriculture and Rural Affairs, Baoding 071000, China; c Hebei Provincial Key Laboratory of Livestock and Poultry Breeding Intelligent Equipment and New Energy Utilization, Baoding 071000, China

**Keywords:** CFD, Poultry house, Aerosol, Thermal comfort, Response surface optimization

## Abstract

In response to the challenges of regulating the thermal environment and the high risk of biological aerosol transmission in closed-scale poultry farming systems, this study focuses on ventilation optimization in poultry houses based on computational fluid dynamics (CFD). First, a comparative numerical simulation was conducted to analyze the airflow characteristics under different fan combination operation modes. The study systematically evaluated the effects of various fan combination schemes on the airflow distribution and biological aerosol diffusion behavior inside the poultry house. Based on these findings, an experimental design was established with fan efficiency, guide vane angle, and inlet temperature as the experimental factors, and the proportion of the thermal comfort zone as the response indicator. A three-factor, three-level response surface simulation experiment was conducted to assess the ventilation process in the poultry house. Subsequently, significance testing and regression equations were established to optimize the parameters. The experimental results indicate that the optimal operational parameters for the ventilation system are: fan efficiency (93%), guide vane angle (9.85°), and inlet temperature (19.09°C), resulting in a maximum thermal comfort zone proportion of 90.59%. Five validation experiments yielded an average thermal comfort zone proportion of 89.02%, with an error of 1.73% compared to the predicted value. The research reveals the impact of fan combination modes on airflow paths in intensive poultry houses and provides a multi-parameter optimization method for environmental regulation. These findings offer theoretical support and technical references for the optimization of environmental control systems in poultry farms.

## Introduction

Temperature, humidity, and air quality within poultry farms play crucial roles in the growth and production of poultry (Boutera et al., 2024). Good air quality can lower the risk of disease transmission among poultry (Uddin et al., 2025), and an appropriate thermal environment supports normal metabolic processes, allowing poultry to maintain optimal growth rates and production efficiencies (Al Amaz and Mishra, 2024).

Air pollutants generated during large-scale farming have a significant negative effect on poultry survival and production (Li et al., 2023; Wang et al., 2023; Zhao et al., 2023). Wang et al. (2024) defined bioaerosols as mixtures of solid or liquid microparticles suspended in the air inside poultry houses, identifying their main sources as the physiological behaviours of poultry and the activities of farm workers. The danger of aerosols in poultry houses lies in their pathogenicity. The longer the pathogenic aerosols remain in the house and the wider their spread, the greater the likelihood of disease transmission (Yang et al., 2024). Du et al. (2019a) and Humbal et al. (2018) summarised the factors influencing the spread of pathogenic aerosols inside poultry houses through experiments, including the geometric shape and layout of the poultry house, mechanical ventilation methods, and poultry behaviours, such as wing flapping, clustering, sneezing, and other activities. Li et al. (2019) suggested that biological aerosols generated during poultry production are mainly expelled from houses through mechanical ventilation. In addition, a small portion of the aerosols may settle and adhere to the walls of the poultry house, from where they are manually removed. A good ventilation system can create effective airflow paths, allowing pathogenic aerosols to be expelled more quickly, thereby reducing the risk of infection (Peña Fernández et al., 2019). Conversely, if the ventilation system is poorly designed or if improper control strategies are used, pathogenic aerosols may spread extensively within the house and remain there for long periods, thereby exacerbating the spread of diseases (Winkel et al., 2015). However, the impact of ventilation systems on aerosol diffusion patterns remains unclear.

Good air quality is fundamental to ensure the normal survival of poultry (Chen et al., 2022). To meet the health, production, and welfare requirements of poultry, suitable thermal comfort conditions must be provided in poultry houses (Ahmadi Babadi et al., 2022; Harrouz et al., 2021; Tong et al., 2019a). Tong et al. (2019a) improved the temperature and humidity heat stress index suitable for poultry farming to evaluate the thermal comfort of poultry houses. Du et al. (2020) developed thermal comfort evaluation criteria based on temperature suitability in poultry farming areas, considering different seasons, poultry species, and farming modes, thereby making the thermal comfort judgment indicators more diverse. Ventilation systems play a crucial role in regulating the thermal environment of poultry houses. Cheng et al. (2018) adjusted the structural design of poultry housing facilities and verified the effectiveness of proper ventilation in regulating the thermal environment through comparative control experiments. Insufficient ventilation can lead to heat accumulation inside houses, resulting in heat stress (Nawaz et al., 2021; Schauberger et al., 2020); which can intensify the wind-chill effect, thereby affecting poultry production (Tong et al., 2019b; Yang et al., 2022).

The typical ventilation system used in poultry houses is tunnel-type negative-pressure ventilation, which requires controlling the airflow entry by adjusting the size and position of the air inlets (Du et al., 2019b; Lee et al., 2022). The ventilation flow rate was controlled by varying the number of exhaust fans and their operating frequencies. In hot summer months, multiple fans work in coordination to adjust the thermal environment, and high-speed airflow effectively expels biological aerosols from the house (Calvet et al., 2010; Wang et al., 2014). Ventilation is particularly challenging during the winter and spring transitional seasons, when a large external temperature difference exists. When the temperature is low, the ventilation rate decreases, leading to the accumulation of biological aerosols inside the house. Therefore, when temperatures increase during the day, a large volume of ventilation must be completed within a short time to ensure proper airflow in a poultry house. Designing a ventilation strategy that ensures good air quality inside a poultry house while maintaining thermal comfort in the environment is an important issue that must be addressed in research on poultry house environmental control.

In poultry house ventilation systems, the fan-on mode exhibits significant variability in the modulation of environmental parameters in the house (Fidaros et al., 2018; Tong et al., 2019a). Differences in the number of fans activated and their operations can lead to the formation of different airflow paths (Choi et al., 2024; Fidaros et al., 2018). During seasons with significant temperature fluctuations and relatively low ventilation rates, the challenge is determining how to activate fans to create the most effective airflow path for regulating the air quality in poultry house. Additionally, theoretical guidelines are lacking on adjusting air inlets and exhaust outlets to achieve an optimal thermal environment regulation strategy under varying external temperatures.

This study combined computational fluid dynamics (CFD) simulations with response surface optimisation to systematically explore the impact of ventilation system components on poultry house environmental parameters. The aim was to reveal the influence of fan operation modes on airflow paths and establish a new method of environmental control based on multiparameter coupled optimisation. This approach provides theoretical support for the precise control of poultry housing environments during seasonal transition periods.

## Materials and methods

**Fig. 1:**
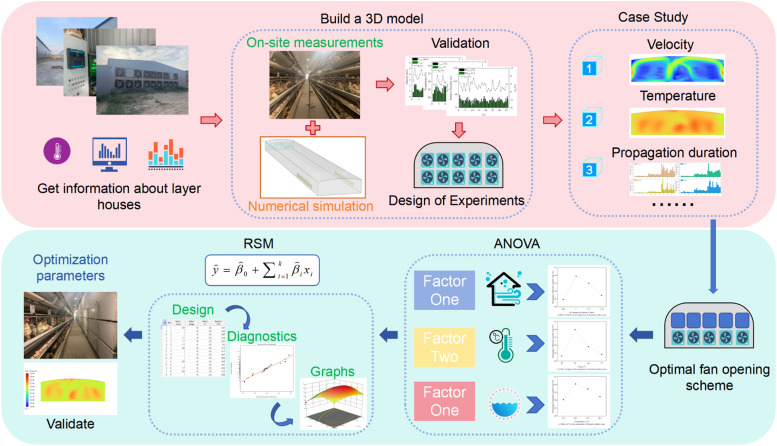
Technology roadmap for optimization research on thermal environment regulation.

This study reduces the risk of pathogen transmission in poultry houses and improves poultry welfare through two steps: simulation modeling and response surface optimization design. The process is shown in (Fig. 1). First, CFD simulations were performed to analyze the impact of different fan combinations on the distribution of environmental parameters within the house and to track bioaerosols. The optimal fan combination was identified, and response surface methodology (RSM) was used for optimization to determine the parameters with the greatest impact on the observed values, such as fan efficiency, air intake angle of the guide vanes, and intake temperature. Finally, detailed thermal comfort parameters in the optimized design were investigated.

### Poultry house

**Fig. 2:**
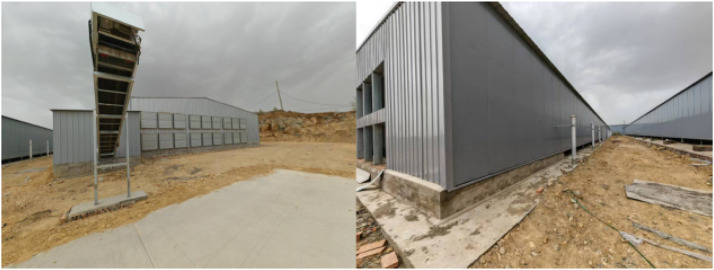
Exterior of the laying hen house.

The experimental building is a factory-style egg-laying poultry house located in the Shahe District of Xingtai City, Hebei Province, China, equipped with fully automated ventilation facilities. The dimensions of the building are 100m in length, 15m in width, and the side wall height is 4.5m, with a maximum distance from the ground to the ceiling of 5.3m. The building structure is shown in (Fig. 2). Inside the poultry house, there are 4 layers and 5 rows of chicken cages, housing approximately 53,280 laying hens with an average weight of 1.9 kg and an egg production rate of about 0.84 eggs per hen per day. The cages are 1.22m in length, 1.22m in width, and 0.40m in height (excluding the manure conveyor). The spacing between each row is 1.48m, and the total height of the four layers of cages is 3.0m, with the bottom of the cages set at a height of 0.50m above the ground.

The automated ventilation control system uses temperature and humidity sensors to collect environmental parameters inside the building. Data is transmitted to the controller via a wired network, which then decides whether to activate the fans. The control logic employs a threshold control method, defining different temperature ranges and corresponding numbers of fans to be activated. The ventilation system's exhaust outlets consist of 18 negative pressure fans with a diameter of 1.4 m and an airflow rate of 38,000 cubic meters per hour, installed in two rows at equal intervals on one side of the end wall. The air intake guide plate of the ventilation system is installed on the front of the two side walls, measuring 21.00 m × 1.50 m, and its opening angle is controlled by hinges.

### Case studies

**Fig. 3:**
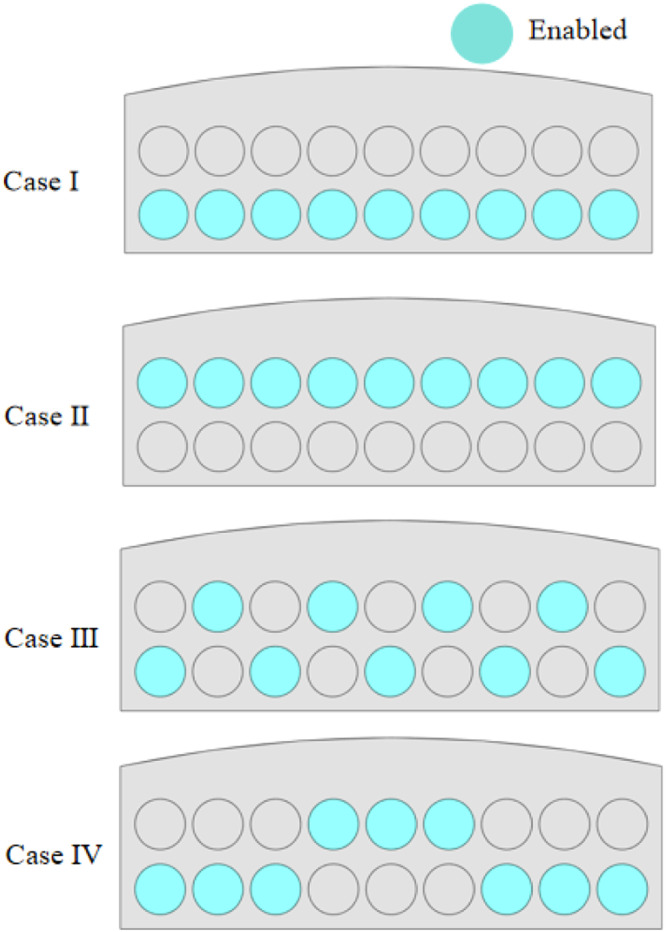
The combination scheme of opening the four sets of fans.

To study the impact of fan operation at different positions on thermal comfort and pathogen dispersion in the poultry house, we selected four classic fan combinations. According to the actual field conditions, nine large fans need to be turned on for ventilation. Case 1: All 9 fans in the first row are turned on; Case 2: All 9 fans in the second row are turned on; Case 3: The 9 fans are alternately turned on; Case 4: Three fans are grouped together, and they are alternately turned on in an up-and-down pattern. The geometric shapes of the four case studies are shown in (Fig. 3).

### CFD simulation

#### Simulation model

**Fig. 4:**
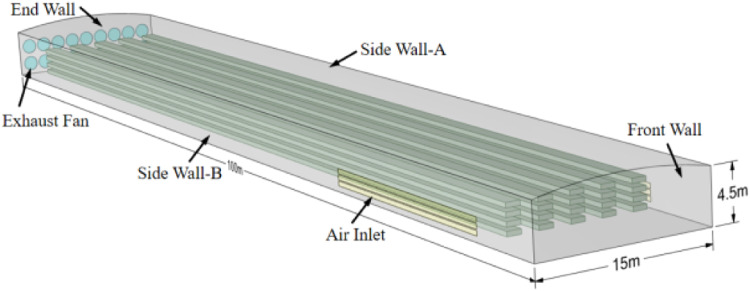
Three-dimensional model of the laying hen house.

In this study, the internal environment of the poultry house was simulated in 3D using the commercial software Fluent V19.0. The geometric model of the poultry house was developed using SpaceClaim, with real dimensions used for modeling (as shown in Fig. 4). The poultry house is oriented east-west, with the southeast corner as the origin of the coordinate system. The positive direction of the X-axis is west, and the positive direction of the Z-axis is north. The experimental area of the poultry house consists of a tunnel structure, including an arched roof, two side walls, the front wall, the end wall, and the floor. The large fans are modeled as circles with a diameter of 1.4m, and their specifications and quantity are based on the actual experimental conditions.

#### Numerical model

The study focuses on the distribution of environmental parameters under ventilation modes, so a steady-state analysis was conducted, and a pressure-based solver was chosen to handle the complex boundary conditions. For the steady-state flow problem, the SIMPLE algorithm was used to handle the coupling between the pressure and velocity fields. The governing equations consist of the continuity equation (Eq. (1)), the momentum equation (Eq. (2)), and the energy equation (Eq. (3)). The RANS *k-ε* turbulence model was selected to evaluate the pathogen trajectories in the air, as it requires far fewer computational resources than large eddy simulation (LES). The standard wall function (SWF) was used for the wall treatment.

(1) $$\frac{\partial \rho }{\partial t}+\nabla \left(\rho \vec{v}\right)=0$$

(2) $$\rho \left(\frac{\partial \vec{v}}{\partial t}+\vec{v}\cdot \nabla \vec{v}\right)=-\nabla P+\nabla \cdot \tau +\vec{f}$$

(3) $$\frac{\partial (\rho e)}{\partial t}+\nabla \cdot \left[\rho \vec{v}\left(e+P/\rho \right)\right]=\nabla \cdot \left(k_{\text{eff}}\nabla T\right)+\Phi +Q$$

where ρ is the fluid density (kg·m^-3^); $\vec{v}$ is the velocity vector (m.s^− 1^); P is pressure (Pa); τ is the viscous stress tensor (Pa); $\vec{f}$is the volume force per unit volume (N·m^-3^); e is the total specific internal energy(m^2^·s^-2^); T is the temperature (K); $k_{\text{eff}}$ is the thermal conductivity (W·M^-1^·K^-1^); Φ is viscous dissipation function (W·m^-3^); Q is external heat source (W·m^-3^).

#### Boundary conditions and particle injection parameters

The airflow speed in the ventilation system is typically low. To more effectively simulate the airflow distribution inside the chicken coop, an incompressible ideal gas model is used. The negative pressure fan is set as the velocity outlet, and the guide vane is set as the pressure inlet. A method of finite position multiple measurements is employed, with an infrared thermographic camera (Testo 890, infrared resolution 640 × 480 pixels, thermal sensitivity < 40 mK) used to measure the temperature of the walls.

The Lagrangian method is chosen to simulate discrete particles because it is more suitable for the dynamics of discrete particles in fluid than the Eulerian method. The movement trajectory of biological aerosols is simulated by considering the changes in particle velocity caused by inertia, gravity, drag, and Brownian motion. The lift of aerosols in the chicken coop is relatively small, so the effect of lift on aerosol propagation is not considered (Du et al., 2019a). Since this study aims to investigate the impact of different fan activation groups on the propagation of biological aerosols, the generation rate and actual release locations of the aerosols are not precisely matched. To minimize the impact on gas flow, a release rate of 1 × 10^-20^ kg/s is used, with the initial velocity of the spray source being the velocity component in the Z-axis direction of that volume node.

A porous media model is used to simulate the chicken activity area (Hu et al., 2024). The effects of the feeding system, drinking system, and egg collection system on the simulation are negligible, so these devices are omitted from the modeling work. During the simulation process, the air guide plate was set as the pressure inlet with a temperature of 24°C, whereas the negative-pressure fan was set as the speed outlet with an outlet velocity of −6 m/s. Detailed boundary condition settings are shown in (Table 1).

**Table 1:** Boundary conditions and injection parameters.

Parameter	Value
Air guide plate	Pressure inlet, 24°C
exhaust fan	Velocity inlet, -6m/s
Discrete term boundary type型	No slip wall, trap
Coop Area	Porous media
Energy source	338.04W/m^3^
Ceiling	No slip wall, 18.3°C
Ground	No slip wall, 10.9°C
Front wall	No slip wall, 15.4°C
End wall	No slip wall, 13.7°C
Side wall-A	No slip wall, 13.9°C
Side wall-B	No slip wall, 13.2°C

#### Meshing and grid independency

In this study, Fluent Meshing grid division software was used to generate the body grid, and the grid elements were divided using a combination of tetrahedral and polyhedral meshes. Fine mesh grids were used in areas close to the walls, air inlets, and air outlets to ensure that the maximum y+ value remained below 5. The gas inside the poultry cage does not require high-resolution boundary layers. Therefore, to reduce computational costs, it was set to a range of 30 to 300. The smaller the grid size, the higher the computational accuracy; however, it also requires more computational resources and longer computation time.

**Fig. 5:**
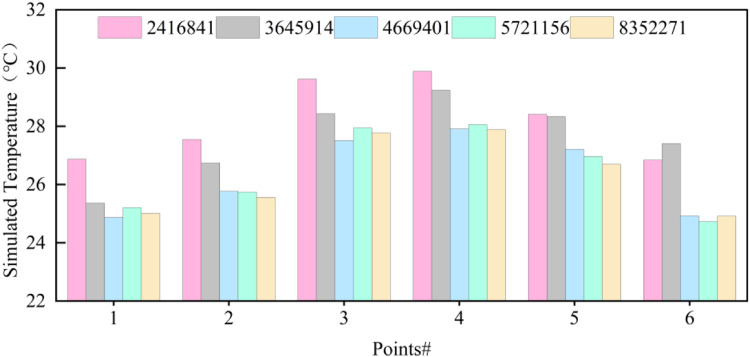
Grid independence test results.

To balance computational resources with computational accuracy, six different specifications were used to study grid independence. The air temperature at six locations was simulated (the selected points were the six locations at the Y=0.8m plane height, as chosen in Section 2.4) to compare the accuracy of the simulation results between different grid sizes (Fig. 5). The grid division was subjected to a grid independency test using the Meshing software.

In order to balance the problem of solution progress and calculation time, the calculation results of 8352271 grid cells are used as the basis to calculate the average relative error of the calculation results of different grid numbers, and the results are shown in (Table 2). According to the results shown in the table, the average relative errors corresponding to the 4669401 grid and 5721156 grid are less than 1% and the difference is not significant, therefore, in order to save the computational time 4669401 grid is chosen as the computational grid in this study.

**Table 2:** Average relative error table.

Number of Grids	2416841	3645914	4669401	5721156
Mean Relative Error	3.12	1.56	0.87	0.94

### Model validation

**Fig. 6:**
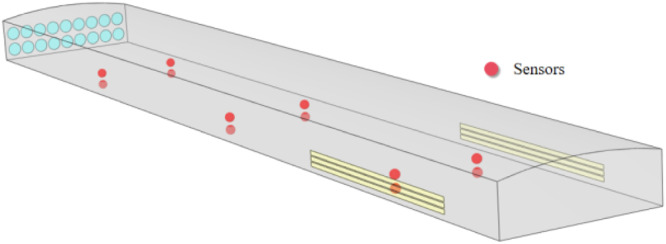
Schematic diagram of sensor measurement position.

To validate the numerical models, 2 heights were measured: (1) the height of the first layer of chicken activities (0.8 m); (2) the middle height of the second and third layer of chicken cages (2 m), which is expected to have a large height of wind speed change, a total of 12 points of temperature results, relative humidity results and air velocity results were compared with the experimental data in this study, (Fig. 6) shows the schematic diagram of the measurement locations. The environmental data were measured using a self-developed wireless sensor network system, including temperature sensors (accuracy ±0.5°C), humidity sensors (accuracy ±3% RH), and air velocity sensors (accuracy ±0.02*v, with v being the true wind speed), and the environmental data were uploaded once every 5s. Since it was not possible to measure all points simultaneously, the environmental conditions outside the house were continuously monitored at three different locations during the measurement period to verify whether the external atmospheric environment changed significantly during the measurement process.

In order to intuitively react to the difference between the data calculated by simulation and the actual measured data, the relative error is used as a test criterion (Du et al., 2019b), and the relative error (Eq. 4) is shown below.

(4) $$E_{r}=\frac{\left|X_{\text{CFD}}-X_{\text{EXP}}\right|}{X_{\text{EXP}}}\times 100\%$$

Where, Er is the relative error, XCFD is the CFD simulated value, and XEXP is the actual measured value.

### Optimization design

#### ANOVA experiments

The poultry house ventilation system includes negative pressure exhaust equipment and an intake building structure. The negative pressure fan is installed on one side of the end wall to exhaust waste gas from the house, creating a negative pressure environment inside. Fresh air from the outside enters through the intake building structure's guide vane, forming the airflow movement path. This process is accompanied by heat exchange and the diffusion of aerosol substances.

Through single-factor experiments, identify the key factors affecting the ventilation system by altering only one variable at a time and observing its impact on the response value, thereby preliminarily determining the influencing factors and their numerical ranges. The exhaust volume, intake area, and intake temperature determine the thermal environment and air quality inside the house. The fan working efficiency (FWE), guide vane opening angle (Angle), and intake temperature (T) are selected as input parameters. Using a variable-frequency drive to adjust the power-supply frequency to the motor, the motor speed can be smoothly adjusted, enabling precise regulation of the fan’s air delivery efficiency. The opening angle of the air deflector was controlled using hinges. The intake temperature in the crotch area was regulated using heating and cooling equipment. The output parameter of the solver is the proportion of the thermal comfort zone (PTCZ). The calculation method for PTCZ is shown in (Eq. 5):

(5) $$PTCZ=\frac{V_{t}}{V_{w}}$$

Where, Vt is the volume of thermal comfort zone and Vw is the volume of effective breeding area of the poultry house. The thermal comfort zone for laying hens is between 18∼25°C (Du et al., 2020). The closer the PTCZ is to 1, the better the ventilation system's control effect is considered.

**Fig. 7:**
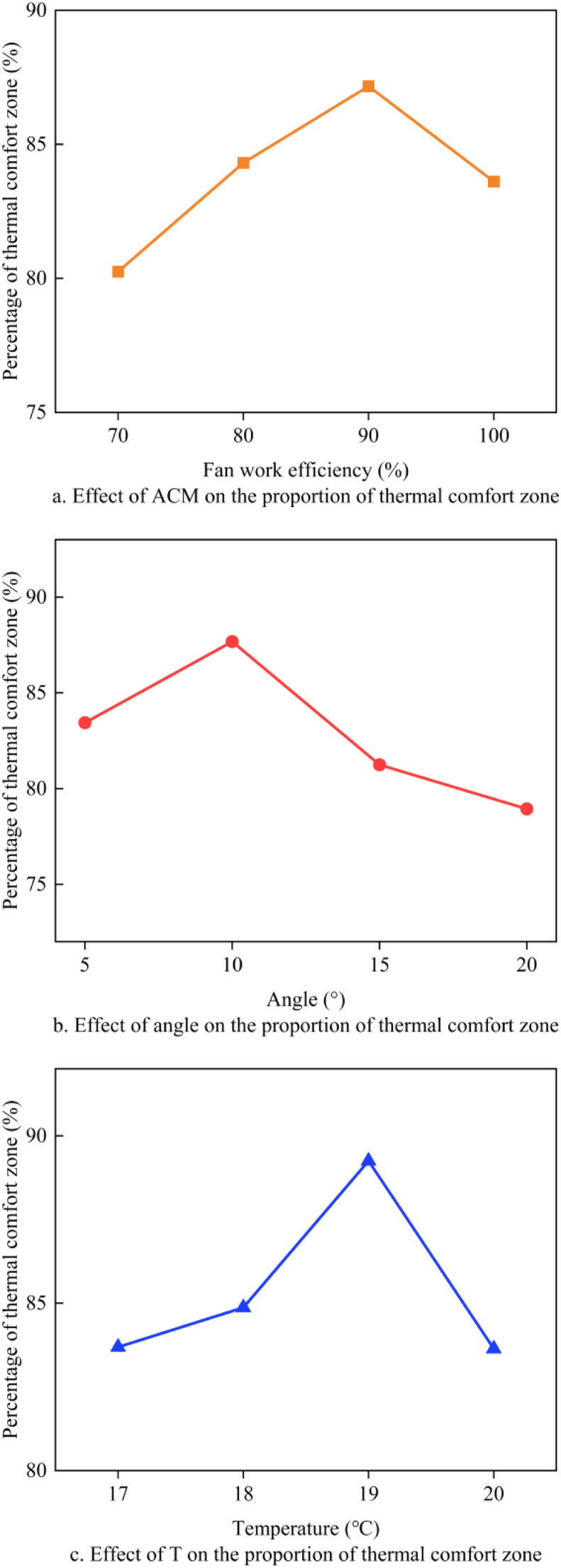
Effect of FWE, Angle and T on the percentage of comfort zone.

ANOVA experiment was conducted to verify the significance differences between input parameters and the proportion of the thermal comfort zone. The test results are shown in (Fig. 7). When the outdoor temperature is below 18°C, the use of longitudinal negative pressure ventilation causes a large area inside the chicken coop to be in a low-temperature zone. When the outdoor temperature exceeds 20°C, some areas experience high-temperature conditions. Based on the results of the single-factor experiment, the ranges of FWE, Angle, and T are defined as (80%∼100%), (5°∼15°), and (18∼20°C), respectively.

#### Response surface optimization experiment

After validating the CFD model, the optimal fan operation mode was determined. Subsequently, the control process of the ventilation system was optimized to improve the comfort index of the laying hens. In ventilation system research, optimizing a single parameter overlooks the interaction effects between parameters, which can easily lead to extreme environmental conditions. Considering all parameters affecting the performance of the ventilation system would require a large number of CFD simulations, which are time-consuming. Therefore, the design of experiments (DOE) method was introduced in the CFD simulation to analyze the interaction between inputs and outputs, thereby reducing the consumption of computational resources.

The optimization process includes three steps: (1) Use the Box-Behnken Design (BBD) method to obtain the design points; (2) Use the Response Surface Method (RSM) to model the relationship between control factors and response results; (3) Obtain the optimal design through the response optimizer.

## Results and discussion

### Numerical model validation

**Fig. 8:**
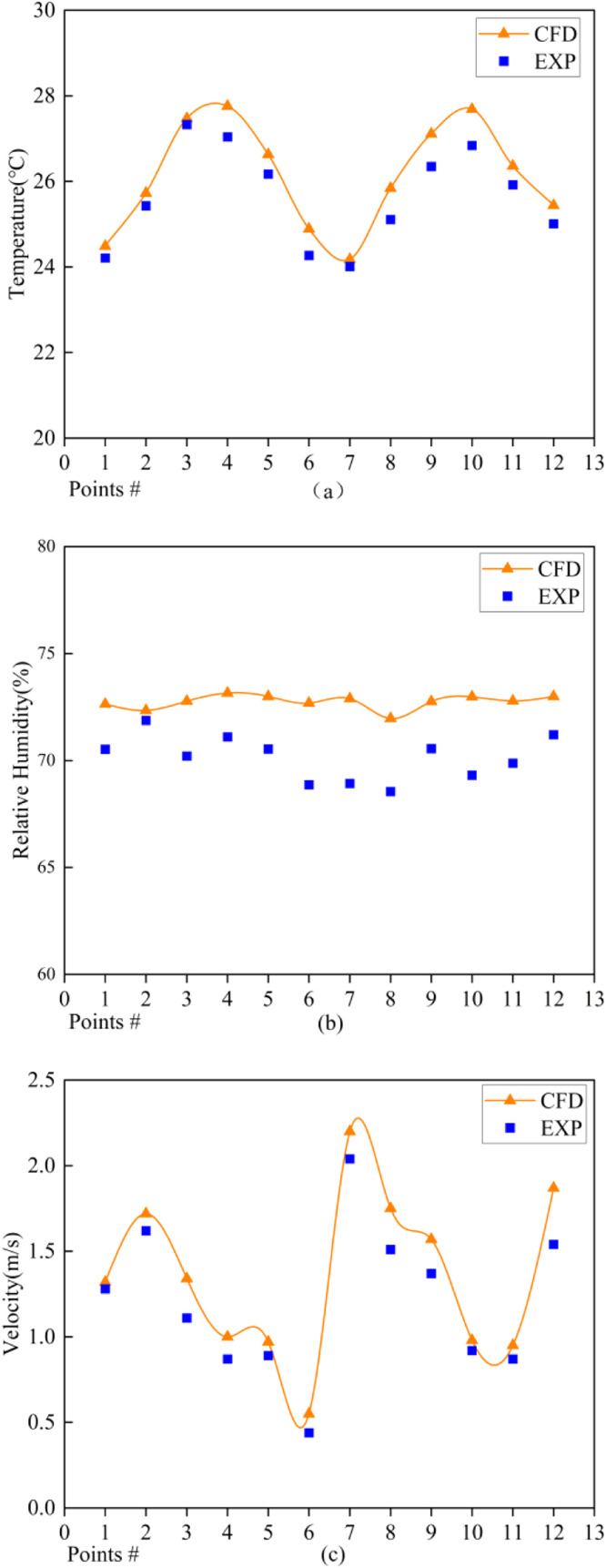
Errors between simulated and measured values (a) temperature error (b) relative humidity error (c) air velocity error.

The CFD simulation values for 12 points at two heights (the height of the chicken flock activity) were compared with actual measurement values, as shown in (Fig. 8). More than half of the temperature and humidity relative errors remained below 5%. The maximum air velocity difference was 0.33 m/s, while the air velocity differences for the other points generally ranged from 0.1 to 0.2 m/s. Larger discrepancies occurred at the top of the chicken coop, as the modeling process ignored automated egg collection equipment and feeding equipment, which led to measurement errors. The predicted values were generally consistent with the experimental measurements, indicating that the chicken coop model is accurate.

The sensitivity experiment results of the turbulence model are shown in (Table 3), which display the average relative errors between the predicted values of temperature and relative humidity and the experimental measurement results under different turbulence models. The *k-ε* RNG turbulence model has the smallest average relative error, so it is used for further numerical simulation studies.

**Table 3:** The different turbulence models test.

Turbulence model	T Error	RH Error
*k-ɛ* RNG	2.39%	3.75%
*k-ɛ* Standard	8.41%	9.22%
*k-ɛ* Realizable	3.58%	4.71%

### Environmental parameter distribution results for case study

In a poultry house with a capacity of 50,000 laying hens, the environmental parameter distribution under four different fan operation combinations was simulated. The temperature data is shown in (Table 4). The results of the four schemes indicate that the maximum temperature differences in the chicken cage areas were 4.48°C, 5.32°C, 6.33°C, and 6.49°C, respectively. The average temperatures were generally equal, with no significant differences. Test results indicate that when the supply air temperature remains constant, changes in the indoor temperature environment correlate solely with the number of activated fans, regardless of their specific locations. The influence of indoor airflow paths on heat dissipation rates is negligible.

**Table 4:** Control group experimental temperature results.

	Minimum °C	Maximum °C	Average °C	Maximum temperature difference °C
Case Ⅰ	24.11	28.59	26.88	4.48
Case Ⅱ	24.11	29.43	26.91	5.32
Case Ⅲ	24.12	30.45	26.89	6.33
Case Ⅳ	24.05	30.54	26.98	6.49

**Fig. 9:**
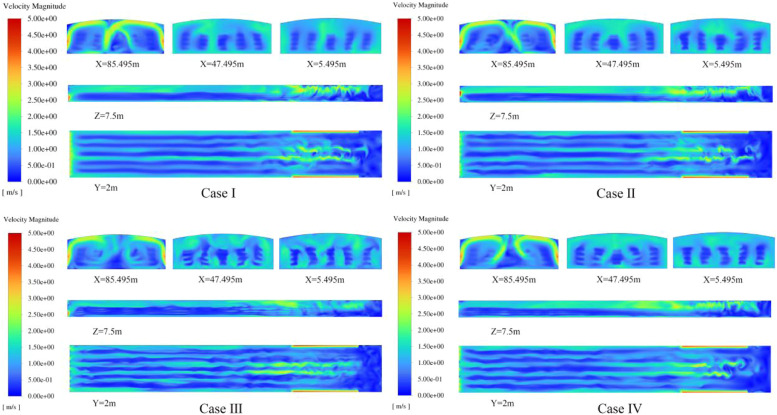
Air velocity distribution in the space region.

The airflow distribution pattern inside the building is shown in (Fig. 9). The average air velocity of Case I, II, and IV is quite similar, remaining above 0.64 m/s, which is higher than the average air velocity of 0.53 m/s in Case III. The air velocity strip distribution diagram for Case III shows significant fluctuations in airflow across various vertical cross-sections. Air flow paths within laying hen houses are influenced by pressure conditions. A regular pressure gradient establishes efficient air flow pathways. Continuously operating fans create a decreasing pressure gradient from the air inlet to the exhaust fan end, accelerating air flow velocity. The intersection of the first and second row of fans results in the expulsion of air from the poultry house, creating an irregular pressure differential inside the house, which leads to irregular airflow movement.

**Fig. 10:**
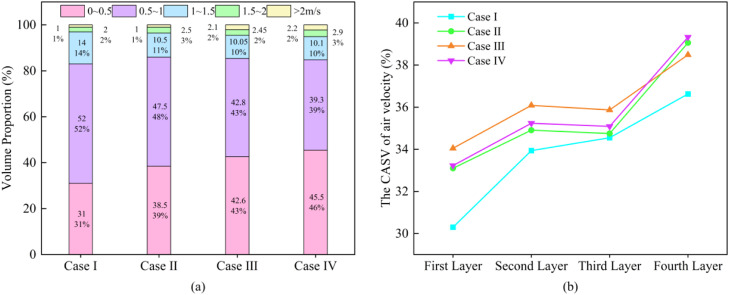
Simulation results of airflow velocity in hen house (a) proportion of air velocity volume in each range of the coop area (b) coefficient of variation of air velocity at different heights.

The proportion of the airflow velocity volume in different ranges for the chicken cage areas is shown in (Fig. 10a). Xie et al. (2024) proposed that the comfortable airspeed range for laying hen houses during the warm season is 0.5∼2 m/s. In Case I, 68% of the area has an airspeed within the comfortable range, which is the largest proportion. For the other three cases, the proportion of the comfortable airspeed area decreases, with a significant portion of the area having airspeed between 0∼0.5 m/s. The airspeed in these regions is too low, which is unfavorable for ventilation in the poultry house. This study uses the airspeed coefficient of variation (CASV) to describe the uniformity of airspeed in different height areas of the chicken cage. The distribution of CASV is shown in (Fig. 10b). In Case I, the CASV at all four heights is smaller than in the other schemes, indicating that prioritizing the operation of the fans closer to the ground results in better uniformity of airspeed distribution inside the house. Case III and IV have larger CASV values, proving that the cross-opening fan method results in poorer airspeed uniformity.

### Results of biological aerosol propagation for the case study

**Fig. 11:**
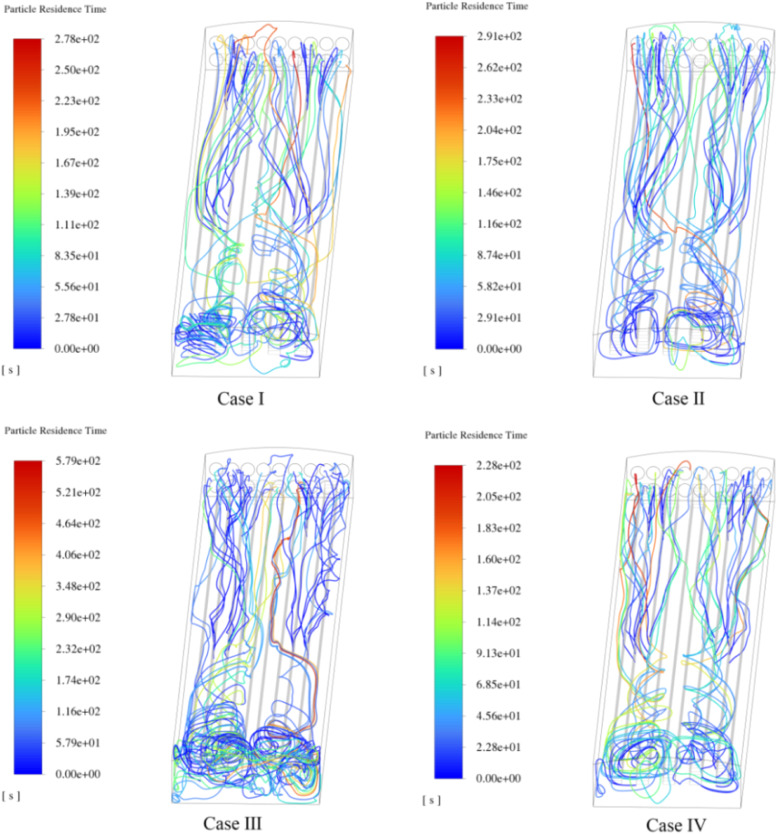
Steady-state airflow streamlines for four cases.

After performing steady-state calculations of the airflow inside the poultry house, the propagation of biological aerosols under four different fan operation combinations was simulated. To better understand the evolution of the aerosol path, 48 injection sources were set up in the front, middle, and rear areas of the chicken coops. (Fig. 11) shows the propagation paths of aerosols exhaled by laying hens. These lines follow the airflow direction, disperse throughout the poultry house, and are eventually expelled by negative-pressure fans. The residence time of aerosol particles is used as a variable to colour the airflow lines, and different fan combination designs have a significant impact on aerosol propagation. Additionally, the airflow lines in Case III were more complex and longer, whereas the airflow paths in Cases I, II, and IV were relatively shorter, reducing the exposure of the poultry. Negative-pressure fans exhaust air from buildings, creating a negative-pressure environment. Driven by the pressure differential, external airflow enters through the intake vents. When adjacent negative-pressure fans operate, their mutual interference is minimal, creating a significant pressure differential between the interior and exterior. This draws the external airflow into the facility at higher velocities, which then flows towards the exhaust fans driven by the pressure gradient. This established an efficient airflow pathway, enabling the expulsion of aerosol particles from the facility within a short timeframe. When nonadjacent fans are operated, a stable pressure gradient cannot be established. Consequently, the external air infiltrates at reduced velocities, leading to a prolonged suspension of aerosol particles within the laying hen house.

**Fig. 12:**
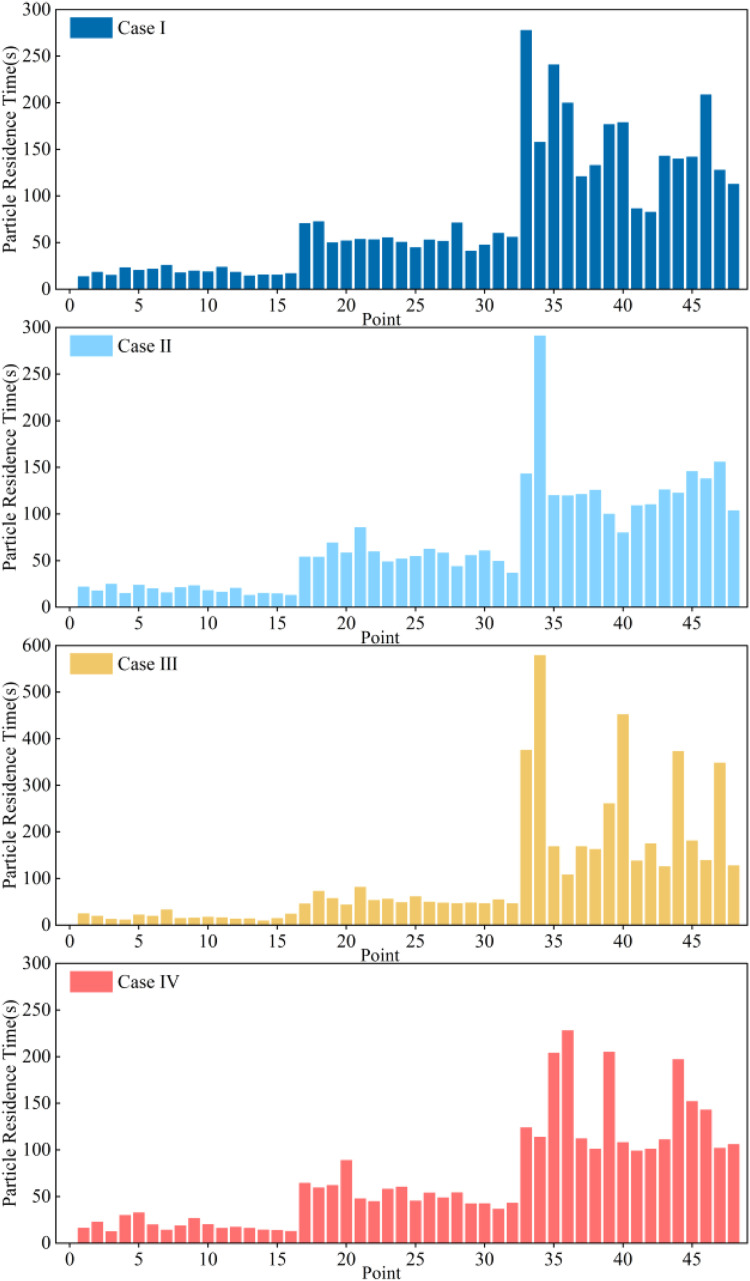
Residence time of exhaled aerosol particles from laying hens.

The residence time of the aerosols inside the poultry house was statistically analyzed (as shown in Fig. 12). Aerosols generated at the end of the chicken coop are typically expelled outside through the negative pressure fan within 60 seconds, with shorter residence times the closer they are to the end. Aerosols released from the injection sources at the front of the chicken coop follow the airflow path and move accordingly. Longer residence times mean that the particles travel through more areas before leaving the poultry house, thus increasing the exposure opportunity for uninfected poultry through the air. It is evident from the figure that a well-designed ventilation system can reduce the residence time of aerosols, and shorter residence times can minimize the risk of contamination spread. The average aerosol residence times for the four cases were 69s, 77s, 104s, and 70s, respectively, with the longest residence times being 278s, 291s, 579s, and 228s. Case III has lower airspeed, and the irregular airflow caused by recirculating airflow results in a higher aerosol residence time. The results show that continuously operating fans in adjacent positions can increase the airspeed, promote heat exchange, and accelerate the expulsion of aerosols.

Comparison of different fan activation schemes on the transmission pathways and residence time of aerosols in poultry houses shows that differences in fan activation locations and the combination of activated fans both influence indoor airflow, thereby affecting indoor air renewal rates and air quality. This experiment confirmed that continuously activating fans at the near-ground end can create more effective airflow pathways, enabling indoor air to be renewed at a higher frequency, accelerating the removal of harmful substances, and ensuring a healthy rearing environment.

### Response surface optimization experiment results

Based on the analysis of the case environment parameter distribution and aerosol propagation results, and in combination with environmental control requirements, the optimal ventilation system was determined by prioritizing the operation of the six fans in the middle positions of the first row near the ground. The system performance was systematically studied by analyzing the impact of three different parameters, FWE, Angle, and T, on the system's performance, and optimization was performed to improve the performance under thermal comfort conditions. In the DOE phase, a set of 15 design points was generated using the BBD technique based on different variables, as shown in the table. All the generated design points were solved in Fluent to evaluate the output parameters. The results of the response surface experiment are shown in (Table 5).

**Table 5:** Design experiment cases.

	Factor 1	Factor 2	Factor 3	Response
RUN	A:FWE(1/min)	B:Angle (°)	C:T(°C)	PTCZ(%)
1	0.83	30.00	24.50	53.01
2	1.17	30.00	24.50	53.21
3	0.83	60.00	24.50	43.12
4	1.17	60.00	24.50	53.34
5	0.83	45.00	23.50	45.65
6	1.17	45.00	23.50	46.33
7	0.83	45.00	25.50	41.27
8	1.17	45.00	25.50	50.2
9	1.00	30.00	23.50	44.32
10	1.00	60.00	23.50	39.21
11	1.00	30.00	25.50	45.11
12	1.00	60.00	25.50	38.96
13	1.00	45.00	24.50	60.41
14	1.00	45.00	24.50	59.87
15	1.00	45.00	24.50	59.55

Multiple regression was fitted to the test results using Design-Expert software to obtain the regression model equation for PTCZ:

$$\begin{array}{c} PTCZ=-3382.8-61FWE+0.26Angle+360.11T-265.5FWE^{2}-0.26472Angle^{2}-9.925T^{2}+5.01FWE\cdot Angle\\ +20.62FWE\cdot T \end{array}$$

**Fig. 13:**
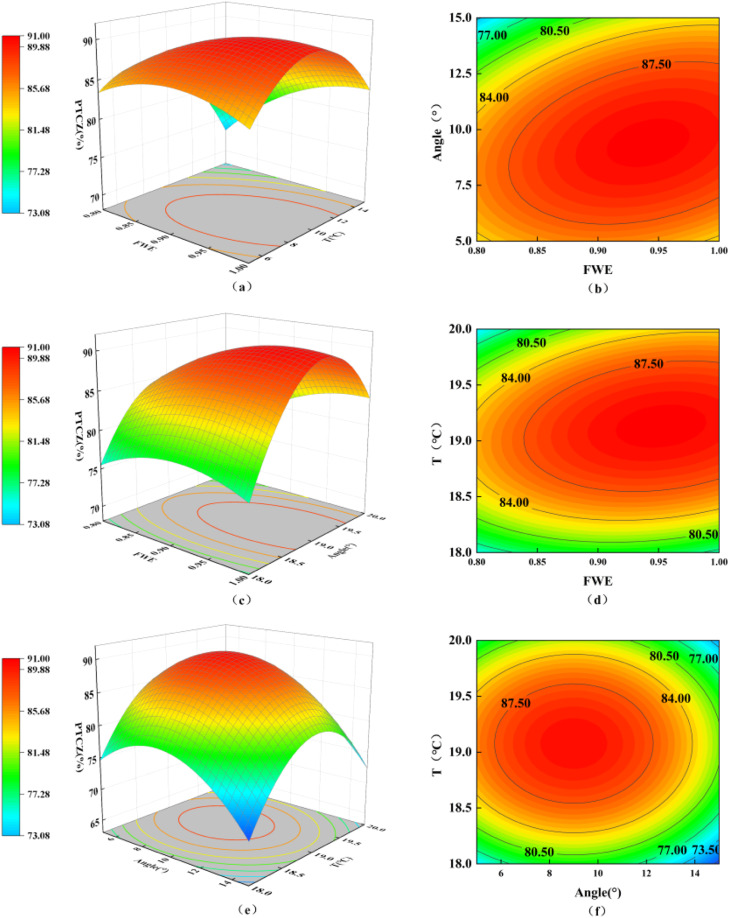
Response surface of poultry house PTCZ with input variables.

The regression model is P<0.01, indicating that the model is highly significant. The value of the misfit term is 0.57 (P>0.05), indicating that the lack of fit is not significant, and the model has a high degree of fit with the experimental results, allowing it to accurately fit the thermal comfort zone proportion (PTCZ). (Fig. 13) describes the interaction of input parameters on PTCZ.In the farming process, the closer the proportion of the comfort zone is to 100% the better. A larger PTCZ value represents higher comfort within the house. The interaction between various independent factors can be explained by the significance of the interaction terms. Under constant temperature conditions, there is a significant interaction between fan working efficiency and guide vane opening angle (AB). Under moderate fan efficiency and an opening angle of 10°, the proportion of the thermal comfort zone is highest. Under the condition where the guide vane opening angle is determined, there is a significant interaction between fan working efficiency and intake temperature (AC). Under moderate fan efficiency and an intake temperature of 19°C, the proportion of the thermal comfort zone is highest. The interaction between the guide vane opening angle and intake temperature (BC) is not significant.

The results indicate that fan efficiency is a key factor in the control of ventilation systems, and its coordinated control with the angle of the deflector plate and the inlet temperature is crucial for optimizing thermal comfort within the barn. Excessive ventilation or changes to the angle of the deflector plate may actually disrupt the uniformity of the thermal environment. These findings provide important theoretical basis and parameter optimization strategies for the precise control of ventilation systems in livestock barns.

### Validation experiment

**Fig. 14:**
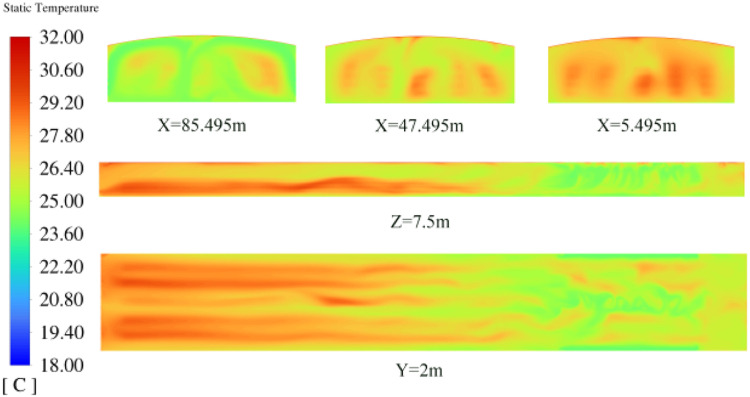
Cloud view of temperature distribution in the space region under optimized control.

The regression model was optimized with the goal of maximizing the thermal comfort zone proportion (PTCZ). Fan working efficiency (FWE), guide vane opening angle (Angle), and intake temperature (T) were used as variables. The optimization results gave the following parameters: fan working efficiency of 93%, guide vane opening angle of 9.85°, and intake temperature of 19.09°C. At this point, the optimized thermal comfort zone proportion is 90.59%. The results of 5 validation experiments are shown in (Table 6), with an average thermal comfort zone proportion of 89.02%, resulting in a prediction error of 1.73%. The third experiment yielded the largest thermal comfort zone proportion, with the results shown in (Fig. 14).

**Table 6:** Validation test results.

	Factor 1	Factor 2	Factor 3	Response
RUN	A:FWE(1/min)	B:Angle (°)	C:T(°C)	PTCZ(%)
1	1.07351	43.2386	24.5407	89.23
2	1.07351	43.2386	24.5407	88.78
3	1.07351	43.2386	24.5407	89.94
4	1.07351	43.2386	24.5407	88.41
5	1.07351	43.2386	24.5407	88.75

The fan working efficiency and guide vane opening angle influence the airflow exchange rate. When the external temperature rises to an appropriate level, ventilation with a fixed airflow exchange rate can effectively expel the waste gases from the house and ensure thermal comfort for the poultry.

## Conclusion

This study utilised the FLUENT simulation software to conduct a three-dimensional steady-state simulation of aerosol dispersion and thermal environmental characteristics within a laying hen house. The Lagrangian method was employed to assess the impact of different ventilation control strategies on particle transport. Orthogonal experiments combined with response surface analysis were used to systematically optimise the ventilation system structure and operational parameters. The primary conclusions are as follows:

(1) Prioritising the activation of fans located near the mid-ground level created an optimal ventilation path, ensuring that 68% of the area maintained airflow speeds within the comfort range. This achieved a minimum temperature difference of 4.48°C and an average aerosol residence time of 69 s. The activation of adjacent fans creates a consistent negative-pressure environment within the laying hen house, establishing effective airflow pathways that facilitate efficient temperature regulation and air renewal within the structure.
(2) The Box–Behnken orthogonal experimental design method was used to study the impact of experimental factors on the proportion of the thermal comfort zone. The relationship between environmental variables and thermal comfort parameters was established through response surface optimisation experiments. The optimal control parameters were determined: fan working efficiency of 93%, guide vane opening angle of 9.85°, and intake temperature of 19.09°C. At this point, the optimal thermal comfort zone proportion is 90.59%. The response surface method was employed to optimise the ventilation system in the laying hen house, fully accounting for the interactive effects of various influencing factors. This approach provides an optimal growth environment for the laying flock while avoiding the waste of resources caused by extensive trial-and-error adjustments within the house.

The results of this study demonstrate that a combination of fan configuration experiments with response surface methodology achieves both efficiency and precision in optimising ventilation systems for livestock and poultry housing. This approach not only helps reduce the risk of aerosol-borne pathogen transmission but also significantly enhances indoor thermal comfort, holding important practical significance for promoting healthy animal husbandry and achieving precise environmental control. Furthermore, by screening trials of key factors in various poultry house ventilation systems, this methodology and its conclusions can be extended to the design and operational optimisation of other poultry house ventilation systems.

## Institutional review board statement

Not applicable.

## Informed consent statement

Not applicable.

## Funding

This project was funded by the National Natural Science Foundation of China (grant no 31902209), Hebei Agriculture Research System (HBCT2024260203) (HBCT2024270208), the Talents Introduction Plan of Hebei Agricultural University under Grant YJ2023049, the research project on Basic Research Business Expenses of Provincial Higher Education Institutions in Hebei Province (KY2024008).

## CRediT authorship contribution statement

**Changzeng Hu:** Conceptualization, Data curation, Formal analysis, Investigation, Methodology, Software, Validation, Visualization, Writing – original draft, Writing – review & editing. **Lihua Li:** Formal analysis, Funding acquisition, Project administration, Supervision, Writing – review & editing. **Yuchen Jia:** Data curation, Formal analysis, Investigation, Project administration. **Zongkui Xie:** Conceptualization, Data curation, Funding acquisition, Methodology, Project administration, Writing – review & editing. **Yao Yu:** Data curation, Investigation, Supervision. **Limin Huo:** Conceptualization, Formal analysis, Funding acquisition, Investigation, Project administration, Supervision, Writing – review & editing.

## Disclosures

The authors declare that they have no known competing financial interests or personal relationships that could have appeared to influence the work reported in this paper.

## Data Availability

The data of this study are available from the corresponding author.
